# Children of mentally ill parents—a pilot study of a group intervention program

**DOI:** 10.3389/fpsyg.2015.01494

**Published:** 2015-10-20

**Authors:** Hanna Christiansen, Jana Anding, Bastian Schrott, Bernd Röhrle

**Affiliations:** ^1^Department of Psychology, Clinical Child and Adolescent Psychology, Philipps-University MarburgMarburg, Germany; ^2^Department of Child and Adolescent Psychiatry and Psychotherapy, Philipps-University MarburgMarburg, Germany

**Keywords:** children, mentally ill parents, prevention, intervention, transgenerational transmission mental disorder

## Abstract

**Objective:** The transgenerational transmission of mental disorders is one of the most prominent risk factors for the development of psychological disorders. Children of mentally ill parents are a vulnerable high risk group with overall impaired development and high rates of psychological disorders. To date there are only a few evidence based intervention programs for this group overall and hardly any in Germany. We translated the evidence based Family Talk Intervention by Beardslee (2009) and adapted it for groups. First results of this pilot study are presented.

**Method:** This investigation evaluates a preventive group intervention for children of mentally ill parents. In a quasi-experimental design three groups are compared: an intervention group (Family Talk Intervention group: *n* = 28), a Wait Control group (*n* = 9), and a control group of healthy children (*n* = 40). Mean age of children was 10.41 years and parental disorders were mostly depressive/affective disorders (*n* = 30), but a small number also presented with Attention-Deficit/Hyperactivity Disorder (*n* = 7).

**Results:** Children of mentally ill parents showed higher rates of internalizing/externalizing disorders before and after the intervention compared to children of parents with no disorders. Post intervention children's knowledge on mental disorders was significantly enhanced in the Family Talk Intervention group compared to the Wait Control group and the healthy control group. Parental ratings of externalizing symptoms in the children were reduced to normal levels after the intervention in the Family Talk Intervention group, but not in the Wait Control group.

**Discussion:** This pilot study of a group intervention for children of mentally ill parents highlights the importance of psycho-education on parental mental disorders for children. Long-term effects of children's enhanced knowledge about parental psychopathology need to be explored in future studies.

## Background

The transgenerational transmission of psychiatric disorders is a major risk factor for the development of mental illness (Mattejat and Remschmidt, 2008; Hosman et al., 2009). According to epidemiological life-time studies, one in five adults will have a mental health problem (Parker et al., 2008; Howard and Underdown, 2011; Rasic et al., 2014). Studies have shown that about 23–32% of patients admitted to mental health care are caring for children (Test et al., 1990; Östman and Hansson, 2002; Fraser et al., 2006; Pretis and Dimova, 2008; Maybery et al., 2009). Given the number of families in Germany and the overall rates of adult psychological illness, this suggests ~3.8 million children that have a parent with a psychological disorder (Wittchen, 2000; Statistisches Bundesamt, 2006; Röhrle and Christiansen, 2009).

Extending the model by Goodman and Gotlib (1999), Hosman et al. (2009) developed a model of the transgenerational transmission of psychological disorders. In this, the four major domains, (a) parent, (b) family, (c) child, and (d) social environment interact with each other and are influenced through five transmission mechanisms: (i) genetics, (ii) prenatal influences, (iii) parent–child interaction, (iv) family influences, (v) social influences outside the family. Further, the model encompasses child development and specific developmental milestones, as well as the concepts of equi-finality (a specific disorder is the result of different causal factors) and multi-finality (a specific risk factor manifests itself through different mechanisms). There is ample evidence confirming assumptions of the model and of the specific postulated risk factors (review Christiansen et al., 2014).

According to meta-analysis direct correlations between parental and child psychopathology are only moderately strong (Connell and Goodman, 2002). An accumulation of additional risk factors that correlate with mental illness, such as low socio-economic status (SES), low quality of life, family conflict, parental stress, parental mental illness as well as child abuse and neglect, and placement outside the home are predictive for increased child disorders (all those factors reviewed in Rutter and Quinton, 1977; Rutter, 1999; Wille et al., 2008; National Research Council and Institute of Medicine, 2009). This holds especially when parental disorders are severe (e.g., psychosis) and genetic factors load strongly—as reflected in high heritability rates (Rasic et al., 2014). Thus, growing up with a mentally ill parent constitutes a major quantitative and qualitative risk for children that is associated with multiple mental and developmental risks in offspring. Examples of such outcomes include a higher infant mortality risk, insecure infant attachment, developmental delays and disorders, internalizing and externalizing disorders, negative long-term outcome and the development of severe psychiatric disorders (review: Wille et al., 2008; Hosman et al., 2009; Greif Green et al., 2010; Kessler et al., 2010; Kersten-Alvarez et al., 2011). This is illustrated in a cross-sectional study by Mattejat and Remschmidt (2008). Here 48.3% of the children and adolescents admitted to psychiatric care had a parent with a mental illness. Garber et al. (2009) showed that offspring of depressed parents are at a two- to three-fold increased risk of developing depressive disorders (Garber et al., 2009). Indeed, longitudinal studies have shown that the life time risk for developing a serious mental illness ranged from 40 to 77% for children of mentally ill parents, and this could be shown for the whole range of psychiatric disorders (review: Hosman et al., 2009). Because psychiatric disorders are a costly problem for society (Greenberg et al., 2003; Lynch et al., 2005; Gladstone and Beardslee, 2009), this highlights the request of the National Research Council and the Institute of Medicine “to shift the focus to advancing health and preventing disorders from occurring in the first place rather than waiting until a disorder is well established and has done considerable harm” (The National Academies, 2009, p. 2).

Various interventions (i.e., cognitive behavior based parent, parent–child, and child intervention programs, mother–infant interaction programs addressing parenting skills, psycho-education programs for parents and children) have been developed to meet the needs of this group of young people (reviews: Fraser et al., 2006; Mattejat and Remschmidt, 2008; Gladstone and Beardslee, 2009; National Research Council and Institute of Medicine, 2009; Röhrle and Christiansen, 2009; Christiansen et al., 2011). Recent reviews of interventions for children of parents with depressive disorders (Beardslee et al., 1998, 2008; Gladstone and Beardslee, 2009; National Research Council and Institute of Medicine, 2009), and children of parents with substance use disorders (Christiansen et al., 2011), have shown positive results with lasting long-term effects (in the Family Talk Intervention studies by Beardslee follow-up effects were sustained for 20 years with children in the intervention group showing lower numbers of psychological disorders). A meta-analysis that included 13 randomized controlled trials demonstrated a relative risk reduction of 40% for children who participated in an intervention, though effects on internalizing (*SMD* = −0.16) and externalizing (*SMD* = −0.22) psychopathology were rather low (Siegenthaler et al., 2012).

One of the most effective programs in the meta-analysis, on which four studies (Siegenthaler et al., 2012) were based, was the Family Talk Intervention. This is a whole-family approach enhancing communication and child resilience (Beardslee et al., 1997a,b,c). One major factor in this program is the communication and the enhancement of knowledge about the parental disorder (Beardslee and Podorefsky, 1988), and both improving communication and resilience have been shown to improve child outcome (Beardslee et al., 1997a,b,c; Solantaus et al., 2010; Punamäki et al., 2013). Core elements of FTI are (1) the assessment of all family members; (2) psycho-education about the parental disorder; (3) connecting the family history to psycho-education about the disorder; (4) reduction of guilt and shame in children; (5) enhancement of child support. This program has also been adapted by other countries, especially in Scandinavia (Solantaus et al., 2010; Pihkala et al., 2011; Punamäki et al., 2013).

In Germany there is both a translation (Beardslee, 2009), and a psycho-analytic adaptation available (Wiegand-Grefe et al., 2012, 2013). We adapted the FTI (Beardslee, 2009) for a group intervention with a total of five children's and two parental sessions, and one individual family session. The employment of a group design enabled us to include more children and we assume that the group experience will contribute to the experience of not being alone with such problems. This approach is based on Yalom's assumption of the universality of dilemmata and has been shown to be a helpful factor in psychotherapeutic interventions (Yalom, 1970).

We expect children in the intervention group to show a reduction in psychopathology and to be more knowledgeable after the Family Talk Intervention group program. The first results of our pilot-study are presented.

## Methods

### Participants

Families were recruited by advertisements in the department of clinical psychology at the University of Marburg. A total of 77 children and their parents participated in the study. Of those, 37 had parents with a mental disorder (*n* = 28 in the Family Talk Intervention group and *n* = 9 in the Wait-List Control group); 40 were children of parents with no disorder (healthy control group). We included the healthy control group to test whether children of mentally ill parents do indeed show higher rates of psychopathology symptoms, and to control for possible maturation effects over time. The mean age of the children was 10.41 years of age (*SD* = 2.66). Due to the department's main research areas, parental disorders were mostly depressive/affective disorders (ICD-10 F3: *n* = 30), but a small number also presented with Attention-Deficit/Hyperactivity Disorder (ICD-10 F90: ADHD; *n* = 7). All parents were initially assessed by senior psycho-therapists who also diagnosed the disorder. Children were referred to our study by adult psychotherapists. A *post-hoc* power analysis established that a total of 66 participants is required to detect small effects in this 3 × 2 design [*F*-test: ANOVA with repeated measures, effect size = 0.25 (see Siegenthaler et al., 2012), α = 0.05, power = 0.95). At baseline there were significant differences between children of parents with mental disorders (Family Talk Intervention group and Wait Control group) and healthy control children (CG) for internalizing and externalizing symptoms rated by questionnaires filled out by the parents (see below), with children of healthy control parents showing overall lower psychopathology ratings. Parents with mental disorders had an overall lower family income (proxy for socio-economic status; SES); there were no significant differences in parental education levels (see Table 1 for details). According to the Declaration of Helsinki the study protocol was approved by the local review board and all parents and children gave written informed consent for study participation.

**Table 1:** **Means, standard deviations (SD) and univariate MANOVA results for age, gender, as well as for mean income and parental education for the three different groups**.

	**Family talk intervention**	**Wait control group**	**Healthy control group**	**MANOVA (univariate results)**
Age	10.57 (2.78)	9.44 (2.55)	10.52 (2.63)	*F*_2_ = 1.37, *p* = 0.26
male/female	12/16	4/5	12/28	*F*_2_ = 0.003, *p* = 0.99
Mean income^*^	2.23 (0.92)	2.00 (0.57)	3.20 (0.82)	*F*_2_ = 8.37, *p* = 0.001
Parental education^**^	2.54 (0.68)	2.28 (0.48)	3.22 (0.76)	*F*_2_ = 0.74, *p* = 0.47

*Mean income is used as a proxy for SES*.

* *1, income 0–15.000 €; 2, income 15.001–40.000 €; 3, income 40.001–65.000 €; 4, income > 65.000 €*.

** *1, no school education; 2, high school; 3, applied sciences; 4, university*.

### Procedure

#### Family talk intervention (FTI)—group version

The Family Talk Intervention group version is based on Beardslee (2009). The Family Talk Intervention program has a total of six sessions with one follow-up session: three parent sessions (two sessions on family history and psycho-education, one for preparing the family session), one child session (psycho-education on the parental disorder), one family session (to enhance communication within the family), and one optional booster session. In our modification of the Family Talk Intervention program, children and parents in this study participated in a small group format (up to five families in one group). The content of the sessions was similar to the original Family Talk Intervention program, with the difference that two double sessions (2 × 90 min) were offered to parents and a total of five sessions for the children (because the group format requires more time). The individual family session was sustained in this program, because this is crucial for the enhancement of parent–child communication (Beardslee, 2009). The optional booster session was not realized in this pilot study. The effectiveness of such interventions in a group setting has been shown for children of depressed parents (Clarke et al., 2001; Garber et al., 2009; review in Christiansen et al., 2011). The total intervention lasted about 3 months. All children were assessed before (pre) and after (post) the intervention. In contrast to the original studies by Beardslee (Beardslee et al., 1993; Beardslee, 2009) there was no long-term follow-up.

### Measurements

#### Child psychopathology

Child psychopathology was assessed before (pre) and after the intervention (post) with either the Child Behavior Checklist (CBCL 4–18; Achenbach, 1991; German adaptation: Döpfner et al., 1994) or the Strength and Difficulties Questionnaire (SDQ; Goodman, 2001). Both scales are rated on a three point Likert scale. Both scales possess satisfying psychometric properties (Döpfner et al., 1994; Goodman, 2001; Woerner et al., 2002), and correlate highly with each other, thus resulting in comparable detection rates of internalizing and externalizing behavior problems (Goodman and Scott, 1999). All parents rated their children's symptoms before and after the intervention with the same scale. The correlation between the CBCL and SDQ total scores in our study was *r* = 0.871 (*p* < 0.001), and Cronbach's alpha in our study was 0.897 for the CBCL and 0.895 for the SDQ We transformed all scores into T-scores to ascertain comparability of test results between the SDQ and CBCL. For the healthy control group 10 CBCL and 30 SDQ ratings were available; for the children of mentally ill parents CBCL ratings were available for all 29 children, and additionally eight children had also SDQ ratings.

#### Knowledge about mental illness

As part of this pilot study, a standardized assessment for the knowledge on mental illness was adapted that has been developed in a prior study (Binnen et al., 2007). According to this, knowledge regarding disorders, emotions, and support possibilities is assessed with 20 items before (pre) and after the intervention (post). Examples of items are “Which psychological illnesses do you know?,” “Who can help when someone has a psychological disorder?,” “How can you recognize that someone is depressed?;” items were answered in a multiple-choice format, i.e., children were given different answering options such as different persons that might or might not help with psychological disorders and were asked to check all the correct ones. The number of correct and incorrect items was calculated as well as the number of correct items minus incorrect ones. The total number of answers results in the total scale from which the two sub-scales (i) knowledge about support and (ii) knowledge about disorders can be derived. Psychometric properties were moderate to satisfactory with test-re-test-reliabilities (6 weeks; Matthias and Siever, 2007) between 0.64 (knowledge about support possibilities) and 0.77 (knowledge about disorders). Cronbach's alpha for the whole scale was is 0.813 and thus the total scale was used for all further analyses. All test scores were z-transformed. All items used can be viewed in the Supplementary Material.

#### Statistical analyses

All data were stored at the department of psychology, Philipps-University Marburg, Germany. All data were analyzed with IBM SPSS Statistics 22. For group comparisons a MANCOVA with repeated measures was calculated controlling for family income, because groups differed significantly on this variable. Since we used both the CBCL and SDQ non-parametric analyses for the subgroup of healthy control children and children of mentally ill parents where both measures were available were performed to make sure that effects could also be obtained for both scales.

## Results

The Levene test was used to test the assumption that variances of the populations from which the samples were drawn were equivalent. This was only significant for internalizing symptoms before the intervention (*p* = 0.002), but not for all the other measures (internalizing symptoms post = 0.052; externalizing symptoms pre = 0.87/post = 0.16; knowledge pre = 0.28/post = 0.69).

**Figure 1:**
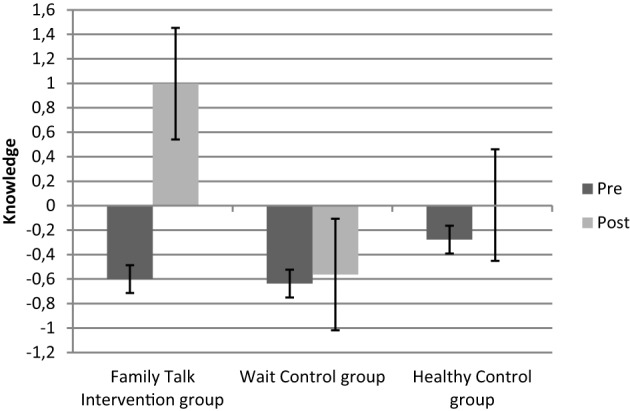
**Z-standardized knowledge-scores before (pre) and after (post) the intervention for the three different groups**.

**Figure 2:**
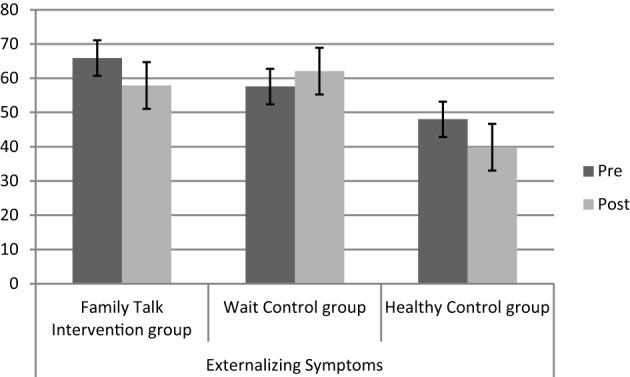
**Levels of externalizing symptoms (T-scores) before (pre) and after (post) the intervention for the three different groups (IG, WCT, CG)**.

**Figure 3:**
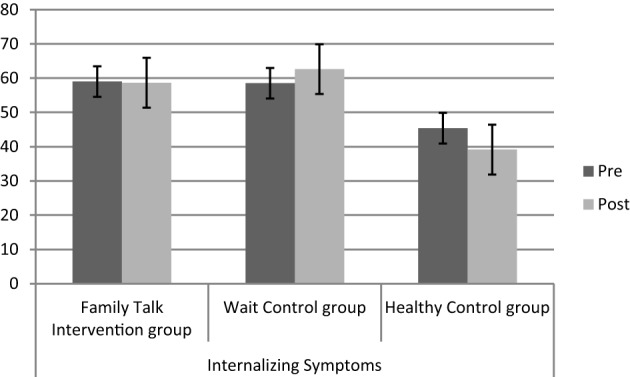
**Levels of internalizing symptoms (T-scores) before (pre) and after (post) the intervention for the three different groups (IG, WCT, CG)**.

The MANCOVA with repeated measures for three groups and two measuring points (pre/post) resulted in a significant effect of group [Wilk's Lambda = 0.32, *F*_(6, 42)_ = 5.06, *p* = 0.001, η^2^ = 0.391], a non-significant effect of time [Wilk's Lambda = 0.90, *F*_(3, 20)_ = 0.709, *p* = 0.55, η^2^ = 0.096], a non-significant effect of family income [Wilk's Lambda = 0.98, *F*_(3, 20)_ = 0.08, *p* = 0.96, η^2^ = 0.013], and a significant interaction group ^*^ time [Wilk's Lambda = 0.37, *F*_(6, 42)_ = 4.21, *p* = 0.003, η^2^ = 0.367]. Univariate results show that the interaction effects were due to changes in knowledge (*F*_2_ = 6.68, *p* = 0.005, η^2^ = 0.378) and externalizing symptoms (*F*_2_ = 4.13, *p* = 0.03, η^2^ = 0.273), but not to changes in internalizing symptoms (*F*_2_ = 2.68, *p* = 0.09, η^2^ = 0.196). As expected, children in the Family Talk Intervention group showed bigger knowledge gains compared to the Wait Control group and healthy control group (mean difference = 0.60, *p* = 0.01), and a reduction in externalizing symptoms (mean difference = 8.87, *p* = 0.03) compared to the Wait Control group, but not for internalizing symptoms (mean difference = 6.10, *p* = 0.22; for details please see Table 2 as well as Figures 1–3).

**Table 2:** **Means and standard deviations (SD) for pre-post knowledge and parent rated internalizing/externalizing symptoms for the three different groups**.

	**Family talk intervention**	**Wait control group**	**Healthy control group**
Knowledge pre	−0.59 (0.20)	−0.63 (0.27)	−0.27 (0.26)
Knowledge post	0.99 (0.26)	−0.56 (0.35)	0.01 (0.34)
Internalizing symptoms pre	59.04 (3.83)	58.55 (5.23)	45.41 (5.08)
Internalizing symptoms post	58.68 (3.55)	62.67 (4.85)	39.16 (4.71)
Externalizing symptoms pre	65.89 (2.79)	57.66 (3.81)	48.00 (3.70)
Externalizing symptoms post	57.88 (3.51)	62.08 (4.79)	39.84 (4.65)

Child psychopathology was assessed with either the CBCL (all children of mentally ill parents and 10 healthy control children were assessed with the CBCL) or the SDQ (30 healthy control children). Non-parametric analyses on these data revealed significant results for the CBCL internalizing pre (Kruskal–Wallis test = 0.044) and post (Kruskal–Wallis test = 0.019) scores, but not for the externalizing ones (Kruskal–Wallis test = 0.38). For SDQ scores significant differences were obtained for internalizing as well as externalizing pre-post scores (Mann–Whitney-U-test < 0.001).

## Discussion

Children of parents with a mental illness in our study showed higher rates of internalizing and externalizing symptoms compared to children of healthy parents. This replicates international results (Beardslee et al., 1998; Clarke et al., 2001; Maybery et al., 2009), and highlights the risk for those children to develop serious mental illness themselves (Hosman et al., 2009).

As expected, children in the Family Talk Intervention group demonstrated significantly more knowledge about parental mental illness, emotions, and support possibilities after the intervention compared to children in the other two groups. The provision of information is one of the major goals for children of mentally ill parents, because this enables the children to evaluate the home situation more adequately, i.e., not to attribute their parent's irritability and aggravation on their behavior, but as symptoms of their parent's illness (Beardslee and Podorefsky, 1988; Lenz and Kuhn, 2011). Based on those studies, we argue that information and knowledge about the parental disorders thus directly results in a reduction of children's feelings of guilt, angriness, and shame which in turn enhances children's self-efficacy. Apart from the reduction of parental disorder symptoms (Wickramaratne et al., 2011), this is one of the major protective factors according to Beardslee and Podorefsky (1988). To estimate such effects with our group intervention program, future studies are necessary that allow the disentanglement of intervention components with differential effects in a longitudinal setting.

Healthy control children and children in the Family Talk Intervention group showed a significant reduction in externalizing symptoms compared to children in the Wait Control group. Children in the healthy control group were already within the normal range before the intervention, thus post-scores might also be due to a floor effect. Psychopathology (internalizing and externalizing symptoms) scores of children in the Wait Control group did not improve, but show a slight increase (see Figure 1). Children in the Family Talk Intervention group reduced their externalizing symptom scores from before to after the intervention to normal levels, which points to a differential intervention effect. However, the fairly short time frame after the intervention should be taken into account. For the evaluation of long-term effects long-term follow-up assessments are necessary that were not realized in this study.

The intervention did not result in a reduction of internalizing symptoms in children of mentally ill parents, although there was a non-significant reduction for the healthy control children. Internalizing symptoms are relatively stable and not very easy to change outside the psychotherapeutic setting (Ferdinand et al., 1995; Beelmann and Lösel, 2007). Even though the power-analysis established a sufficient power to detect small effects, it is likely that the time frame to detect such effects was too short in this case: the post assessment following the intervention lasted ~3 months. A long-term follow-up assessment might produce different results, since other studies demonstrate a significant reduction in internalizing symptoms after 9–15 months (Clarke et al., 2001; Beardslee et al., 2008), and externalizing symptoms for the Family Talk Intervention group decreased in the short time frame.

Further, parents with a mental illness have lower incomes in our study compared to the healthy parents. This is also in line with studies showing that low socio-economic status (SES) is associated with higher rates of mental disorders—both in adults and children (Costello et al., 2003; Huss et al., 2008; Wille et al., 2008; Marmot-Report et al., 2010).

## Limitations

We were not able to randomly assign children to the different groups. Future studies should be randomized controlled trials with balanced group sizes and a long-term follow-up to establish possible long-term intervention effects. The original Family Talk Intervention also includes booster-sessions that are not realized in this study, but might contribute to long-term effects. This should be considered in future studies.

Further, even though children of mentally ill parents have overall higher internalizing/externalizing symptom scores, groups are too small for sub-analysis comparing children with high vs. low symptoms.

A separate analysis of knowledge about support and knowledge about disorders would have been preferable. Since Cronbach's alpha of the support scale was low (0.64) we used the whole knowledge scale (Cronbach's alpha = 0.81) instead. Psychometric improvement of the sub-scales is desirable.

Our study included only one type of intervention. Thus, we cannot conclude that the effects of the intervention were exclusively the result of the therapeutic method and its content. The intervention setting might also have had an effect. In future studies, a comparison with a brief or minimal intervention might shed light on this.

When we planned the study, we wanted to use the CBCL for a comprehensive assessment of possible psychological impairments. However, as the CBCL is quite long we decided to also include the SDQ in order to lower thresholds for study participation for the healthy control group. Both scales correlate highly with each other and have been shown to detect rates of internalizing and externalizing behavior problems equally well (Goodman and Scott, 1999). The greater number of items in the CBCL creates a greater number of opportunities to observe and record change which in the case of the decline in externalizing symptoms might reflect regression to the mean. But, a separate non-parametric analysis comparing children of both groups with data available on both scales confirmed the significant differences between groups. Even though, in future studies all participants should be assessed with the same measure to avoid the problem of measurement non-equivalence.

## Conclusion

Even though only a pilot study this is one of the largest studies in Germany so far. There are various projects for children of mentally ill parents in Germany, but the majority are not evidence based (Christiansen et al., 2014). Bearing in mind the large number of adults faced with mental illness (Wittchen et al., 2011) as well as the trans-generational transmission of mental disorders (Hosman et al., 2009), there is an urgent need for the development of effective interventions for this high risk group. Enhancement of knowledge about the parental disorder might prove to be effective for this aim, but this needs to be confirmed in future studies.

### Conflict of interest statement

The authors declare that the research was conducted in the absence of any commercial or financial relationships that could be construed as a potential conflict of interest.
