# Supplementary material for: Children of mentally ill parents—a pilot study of a group intervention program
Source: Front Psychol. 2015 Oct 20;6:1494. doi: 10.3389/fpsyg.2015.01494 (PMC4611090; doi:10.3389/fpsyg.2015.01494)
Supplement: Supplementary file 1 [file DataSheet1.DOCX]

**Appendix**

Items of the knowledge scale used

1. List of different physical illnesses (children list all disorders they know and correct ones are coded)
2. List of psychological illnesses (children list all disorders they know and correct ones are coded)
3. How do you know that someone has a psychological disorder/illness (i. e. symptoms listed # correct MC answers)
4. Who can help when someone has a psychological disorder/illness (i. e. different professions listed # correct MC answers)
5. How can a specialist help when someone has a psychological disorder/illness (i. e. different interventions listed # correct MC answers)
6. Is it okay to be cross with your parents sometimes? (i. e. different examples with different answering options listed # correct MC answers)
7. What is the name of the hospital where psychological disorders/illnesses are treated? (i. e. different institutions listed # correct MC answers)
8. Are psychological disorders/illnesses contagious? (i. e. different examples with different answering options listed # correct MC answers)
9. What do people do in psychotherapy? (i. e. different examples listed # correct MC answers)
10. Whose fault is it when parents become psychologically ill? (i. e. different examples listed # correct MC answers)
11. Who will help children when they have problems (i. e. are sad…) (i. e. different professionals listed # correct MC answers)
12. What does confidentiality mean? (i. e. different explanations listed # correct MC answers)
13. What is a psychiatry? (i. e. different explanations listed # correct MC answers)
14. Can parents get a psychological disorder when children tease them? (i. e. different examples listed # correct MC answers)
15. What emotions do you know? (i. e. different emotions listed # correct MC answers)
16. Is there a need to be ashamed of psychological disorders? (i. e. different examples listed # correct MC answers)
17. Can anybody get a psychological disorder?
18. Is it okay for children to have the need to be alone? (i. e. different examples listed # correct MC answers)
19. What is the child and youth telephone? (i. e. different examples listed # correct MC answers)
20. Can psychological disorders get better? (i. e. different examples listed # correct MC answers)

* MC = multiple choice
